# The Modulatory Role of Orexin 1 Receptor in CA1 on Orofacial Pain-induced Learning and Memory Deficits in Rats

**DOI:** 10.18869/nirp.bcn.8.3.213

**Published:** 2017

**Authors:** Razieh Kooshki, Mehdi Abbasnejad, Saeed Esmaeili-Mahani, Maryam Raoof

**Affiliations:** 1. Department of Biology, Faculty of Sciences, Shahid Bahonar University of Kerman, Kerman, Iran.; 2. Laboratory of Molecular Neuroscience, Neuroscience Research Center, Institute of Neuropharmacology, Kerman University of Medical Sciences, Kerman, Iran.; 3. Department of Endodontics, School of Dentistry, Kerman University of Medical Sciences, Kerman, Iran.

**Keywords:** Orofacial pain, Orexin 1 Receptor, CA1, Learning and memory, Capsaicin

## Abstract

**Introduction:**

Cognitive impairment is commonly associated with pain. The modulatory role of *orexin 1 receptor* (OX1R) in pain pathways as well as learning and memory processes is reported in several studies. The current study was designed to investigate the possible role of CA1-hippocampal OX1R on spatial learning and memory of rats following capsaicin-induced orofacial pain.

**Methods:**

Orofacial pain was induced by subcutaneous intra lip injection of capsaicin (100 μg). CA1 administration of orexin A and its selective antagonist (SB-334867-A) were performed 20 minutes prior to capsaicin injection. Learning and spatial memory performances were assessed by Morris Water Maze (MWM) task.

**Results:**

Capsaicin treated rats showed impairment in spatial learning and memory. In addition, pretreatment with orexin A (20 and 40 nM/rat) significantly attenuated learning and memory impairment in capsaicin-treated rats. Conversely, blockage of OX1R via SB-334867-A (40 and 80 nM/rat) significantly exaggerated learning and memory loss in capsaicin-treated rats.

**Conclusion:**

The obtained results indicated that CA1 OX1R may be involved in modulation of capsaicin –induced spatial learning and memory impairment.

## 1. Introduction

Cognitive impairment is observed in patients with pain, especially chronic pain (Hart, Martelli, & Zasler, 2000; Moriarty, McGuire, & Finn, 2011). Several studies attempted to characterize anatomical and functional brain changes in patients with pain. Orofacial pain entities, covering any pain which is felt in facial and mouth structures, are often very common and complex (Macfarlane et al., 2002; Sessle, 1999). The trigeminal nerve conveys most pain impulses from the orofacial region (Capra & Dessem, 1992). Although several researches support the association between chronic pain and impaired cognitive function, a small portion of them are focused on orofacial pain. Two studies recently showed that capsaicin-induced pulpal pain is associated with impairment of learning and memory, and neuronal apoptosis in hippocampus of rats (Raoof et al., 2015a; Raoof et al., 2015b).

Orexin neuropeptides (A and B), produced by lateral hypothalamus neurons, are agonists for 2 Gq-protein coupled receptors, OX1R and OX2R (Sakurai et al., 1998). Orexin receptors (OXRs) are diffusely distributed in various areas of the brain and implicated in the regulation of multiple physiological functions (Marcus et al., 2001; Peyron et al., 1998; Van Den Pol, 1999). Hippocampus formation is an important target receiving strong orexinergic fibers’ inputs, suggesting that orexin may contribute to processing of memory (Jaeger et al., 2002; Peyron et al., 1998; Trivedi et al., 1998). Blockade of CA1 OX1Rs leads to impairment in spatial memory in rats (Akbari, Naghdi, & Motamedi, 2006). Moreover, blockade of dentate gyrus (DG) OX1Rs is associated with decreased occurrence of long-term potentiation (LTP) in the DG granular cells (Akbari, Motamedi, Davoodi, Noorbakhshnia, & Ghanbarian, 2011).

Aou et al. (2003) reported that intracerebroventricular (i.c.v.) administration of orexin A induced spatial memory deficient in Morris Water Maze (MWM) and resulted in a significant impairment of LTP in the hippocampal CA1 area. In addition, orexin fibers densely project to pain transmission pathways in the brainstem, spinal cord, periaqueductal gray, thalamus, and hypothalamus (Peyron et al., 1998; Van Den Pol, 1999). It is shown that systemic and central administration of orexin A can suppress nociceptive inputs at either spinal or supraspinal levels (Bingham et al., 2001; Holland, Akerman, & Goadsby, 2006; Yamamoto, Saito, Shono, Aoe, & Chiba, 2003). Hippocampal formation indirectly receives trigeminal inputs from preoptic area, orbitofrontal cortex, and amygdale (Burstein & Giesler, 1989).

Especially, it is shown that orexin A microinjection into trigeminal nucleus caudalis can attenuate capsaicin-induced orofacial pain as well as pain-induced learning and memory deficiency on MWM performance (Kooshki, Abbasnejad, Esmaeili-Mahani, & Raoof, 2016). However, the role of hippocampal OX1Rs in orofacial pain-induced learning and memory impairment is not fully clarified. Therefore, the current study aimed at finding the effect of OX1Rs agonist (orexin A) and antagonist (SB-334867-A) microinjection into hippocampal CA1 on spatial learning and memory in the rat model of orofacial pain.

## 2. Methods

### 2.1. Animals

Adult male Wistar rats, weighed 230 to 270 g, purchased from animal house of Shahid Bahonar University of Kerman were used in the current study. The rats were housed under controlled the conditions (23±1°C; 12:12 hours light/dark cycle). Rats had free access to food and water ad libitum. The Ethical Committee of Kerman University of Medical Sciences approved the protocols used in the current study.

### 2.2. Surgery

Rats were anesthetized with a mixture of ketamine (100 mg/kg) and xylazine (2.5 mg/kg). Afterwards, animals were placed in a stereotaxic apparatus and two 22-gauge stainless steel guide cannulae were bilaterally implanted in the CA1 region according to the Paxinos and Watson Atlas of the rat brain (3.8 mm posterior to the bregma, 2.2 mm lateral from the midline and 3.2 mm depth to the cortical surface). Cannulas were fixed to the skull surface with 2 small screws and dental cement. The cannulas were, then, closed with a stylet. After surgery, animals were housed individually and allowed at least 1 week to recover from surgery prior to drug administration and behavioral experiments.

### 2.3. Drugs

Capsaicin was purchased from Sigma-Aldrich, USA, and dissolved in ethanol/Tween 80/distilled water (1:1:8). Orexin A and SB-334867-A were purchased from Tocris Co. (USA). Orexin A was dissolved in distilled water. SB-334867-A was dissolved in dimethyl sulfoxide (DMSO). The final concentration of DMSO was less than 0.1%.

### 2.4. Microinjection

Intra-CA1 microinjection of orexin A and SB-334867-A were performed using 27-gauge needle attached to a 1 μL Hamilton micro-syringe. The injection needle was inserted 1 mm beyond the tip of the guide cannula and infusions were delivered in a total injection volume of 2 μL (1 μL each side) during 1 minute. After each infusion, the needle was remained in the place for 30 seconds before it was slowly retracted.

### 2.5. Experimental design

Rats were divided into 6 groups (n=6) as follows: capsaicin vehicle group (Caps vehicle), which received vehicle of capsaicin; capsaicin-treated group (Caps), which received subcutaneous injections of capsaicin (100 μg) into the upper lip; capsaicin+orexin A (Caps+OXA) treated group, which received intra–CA1 microinjections of orexin A (20 and 40 pM) 20 minutes prior to injection of capsaicin; and capsaicin+SB-334867-A (Caps+SB) treated group, which received intra-CA1 microinjections of SB-334867-A (40 and 80 nM) 20 minutes prior to capsaicin injection, capsaicin plus orexin A vehicle (Caps+OXA vehicle) group, which received distilled water as orexin A vehicle (Shu et al., 2014), and capsaicin plus SB-334867-A vehicle-treated (Caps+SB vehicle) group, which received DMSO as SB-334867-A vehicle.

On the examination day, animals were taken to the testing room at least one hour prior to the experiments. Orofacial pain was induced by subcutaneous injection of capsaicin into the left upper lip in a volume of 10 μL just lateral to the midline and the nose of the rat with a 30 gauge hypodermic needle. After capsaicin injection, the rats were placed in Plexiglass observation chambers, with a mirror located at a 45° angle beneath the floor allowing unhindered observation of the rats. Time of typical pattern of face rubbing was used to assess nociceptive behaviors as previously descripted (Kooshki et al., 2016). Twenty minutes after capsaicin injection, learning and memory performances were considered by MWM test (Morris, 1984).

### 2.6. Morris Water Maze Test

The water maze apparatus comprised of a black circular tank. The pool was divided into 4 equal quadrants defined by the cardinal directions (N, E, S, and W). The pool was filled with water (20±1°C) to a depth of 25 cm. A hidden circular platform (11 cm diameter) was located in the center of one of the quadrants 2 cm beneath the water surface. Cues, consisted of geometric images, were hung on the walls of the test room and were visible to the rats. The position of each rat was monitored by a video camera and analyzed using computerized tracking system. A day before the experiment, rats were allowed to swim for 60 seconds without the platform for habituation.

#### 2.6.1. Acquisition Test

The acquisition test protocol consisted of 4 blocks (4 trials per block). In each trial, the rats were allowed to swim for 60 seconds to find the hidden platform. If a rat could find the platform, the animal was allowed to stay 30 seconds on it. However, if a rat failed to find the platform within 60 seconds, the animal was manually guided to the platform. There was a 5-minute interval between the trials in a block and a 20-minute rest between the blocks. The rats’ escape latency, traveled distance, and swimming speed to reach the hidden platform in each block were recorded (average of 4 trials). In addition, the main latency, travelled distance, and swimming speed in all blocks (including 16 trials) were considered.

#### 2.6.2. Probe Test

A single probe trial was performed 2 hours after the last training trial to test the spatial memory and retrieval capabilities of platform position. In this trial, the platform was removed from the maze. Animals were placed in the quadrant opposite the target quadrant and were allowed to swim freely for 60 seconds. The total time spent in the goal quadrant and swimming paths during the probe trial were recorded.

### 2.7. Statistical analysis

Data related to the 4 training blocks from acquisition test were analyzed by repeated-measures ANOVA. In addition, data from each block of hidden platform tests and probe training sessions were analyzed by one-way ANOVA. Post hoc analysis was performed using the Tukey test and the significance level was set at P<0.05.

## 3. Results

### 3.1. Acquisition Test

#### 3.1.1. Latency time

**Figure 1.**
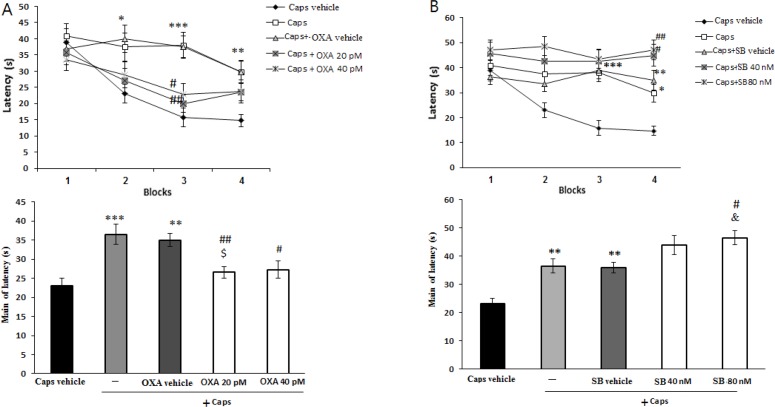
Effects of pre-training intra-CA1 administration of orexin-A (A) and SB-334867-A (B) on escape latency in capsaicin-treated rats. Mean escape latency in each block (upper graph) and in 4 blocks (lower graph) are shown. Values correspond to mean±SEM (n=6), ***P<0.001, **P<0. 01, *P<0.05 versus Caps vehicle group, ^##^P<0.01, ^#^P<0.05 versus capsaicin-treated group (Caps), ^$^P< 0.05 versus Caps+ OX-A vehicle group, ^&^P<0.05 versus Caps+SB vehicle.

Figure 1 A shows the effects of orexin A administration on escape latency of capsaicin-treated rats. The results of repeated-measures ANOVA showed significant effects of treatment [F(4, 115)=8.023; P=0.001] and blocks [F(3, 345)=15.7; P=0.001]. In the capsaicin-treated group, the escape latency significantly increased in the second (P<0.05), third (P<0.001), and fourth (P<0.01) blocks of acquisition test as compared to that of the vehicle group. However, the effect of capsaicin on escape latency in the third block of the acquisition test significantly attenuated by microinjection of orexin A 20 and 40 pM (P<0.01 and <0.05, respectively) (Figure 1A, upper graph). In addition, capsaicin-treated rats showed a significant increase in the mean escape latency compared with the vehicle group (P<0.001) (Figure 1A, lower graph). Moreover, administration of orexin A at 20 pM (P<0.01) and 40 pM (P<0.05) significantly decreased the escape latency in capsaicin-treated rats (Figure 1A, lower graph). Figure 1B illustrates the effect of SB-334768-A administration on escape latency during acquisition test in capsaicin-treated rats.

There were significant differences in treatment [(F(4, 115)=13.549; P=0.001)] and blocks [(F(3, 345)=4.647; P=0.003)]. In capsaicin–treated rats, SB-334867-A at doses of 40 nM (P<0.05) and 80 nM (P<0.01) enhanced the escape latency to find the platform during the block 4 of acquisition testing (Figure 1B, upper graph). Moreover, pretreatment with SB-334867-A (80 nM) significantly increased the mean escape latency in capsaicin-treated rats (P<0.05) (Figure 1B, lower graph).

#### 3.1.2. Traveled distance

**Figure 2.**
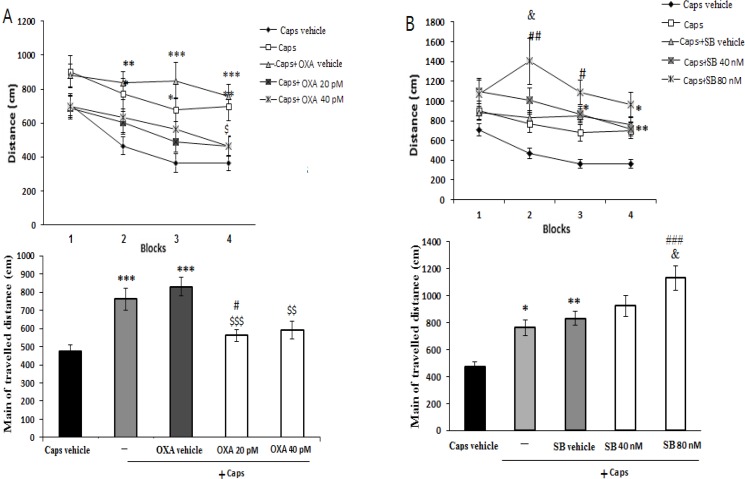
Effects of pre-training intra-CA1 administration of orexin-A (A) and SB-334867-A (B) on traveled distance in capsaicin-treated rats. Mean traveled distance in each block (upper graph) and in 4 blocks (lower graph) are shown. Values correspond to mean±SEM (n=6), ***P<0.001, **P<0.01, *P<0.05 versus Caps vehicle; ^###^P<0.001, ^##^P<0.01, ^#^P<0.05 versus capsaicin-treated group (Caps), ^$$$^P<0.001, ^$$^P<0.01, ^$^P<0.05 versus Caps+OX-Avehicle, ^&^P<0.05 versus Caps+SB vehicle.

Figure 2 A shows the effects of orexin A microinjection on the distance traveled by rats to find the hidden platform. Significant effects of treatment [F(4, 115)=9.912; P=0.001)] and blocks [F(3, 345)=11.688; P=0.001)] were observed. Capsaicin-treated rats traveled significantly greater distances to find the hidden platform than the ones in the vehicle group (P<0.001) (Figure 2A, lower graph). During block 4 of the acquisition trial, the administration of orexin A (20 pM) significantly decreased the traveled distance as compared with that of the capsaicin group (P<0.05) (Figure 2A, upper graph).

In addition, the overall distance traveled by rats with pain pretreated with 20 pM orexin A significantly decreased as compared to that of capsaicin-treated group (P<0.05) (Figure 2A, lower graph). Pre-treatment with SB-334867-A (80 nM) significantly increased traveled distance to find the hidden platform in blocks 2 and 3 as compared to that of capsaicin-treated rats (P<0.01 and <0.05, respectively) (Figure 2B, upper graph). Furthermore, an increase in total traveled distance was observed following capsaicin-plus SB-334867-A (80 nM) administration (P<0.001) (Figure 2B, lower graph).

#### 3.1.3. Swimming speed

**Figure 3.**
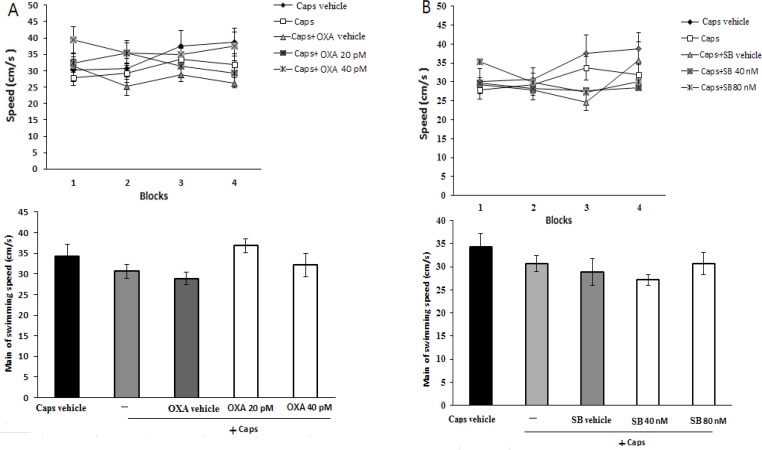
Effects of pre-training intra-CA1 administration of orexin A (A), and SB-334867-A (B) on swimming speed in capsaicin-treated rats; Mean of swimming speed in each block (upper graph) and in 4 blocks (lower graph) are shown. Values correspond to mean±SEM (n=6).

As shown in Figure 3A, administration of orexin A (20 and 40 pM) did not significantly change swimming speed within acquisition trial blocks [F (3, 345)=0.838; P=0.474] (Figure 3A, upper graph) and the mean swimming speed [F(4, 115)=1.998; P=0.099] in capsaicin-treated rats (Figure 3A, lower graph). Likewise, pre-treatment with SB-334867-A (40 and 80 nM) had no significant effect on the mean of swimming speed [F(4, 115)=1.558; P=0.19] (Figure 3B, lower graph), and swimming speed during each block of acquisition test [F(3, 345)=0.674; P=0.579] (Figure 3B, upper graph).

### 3.2. Probe Test

**Figure 4.**
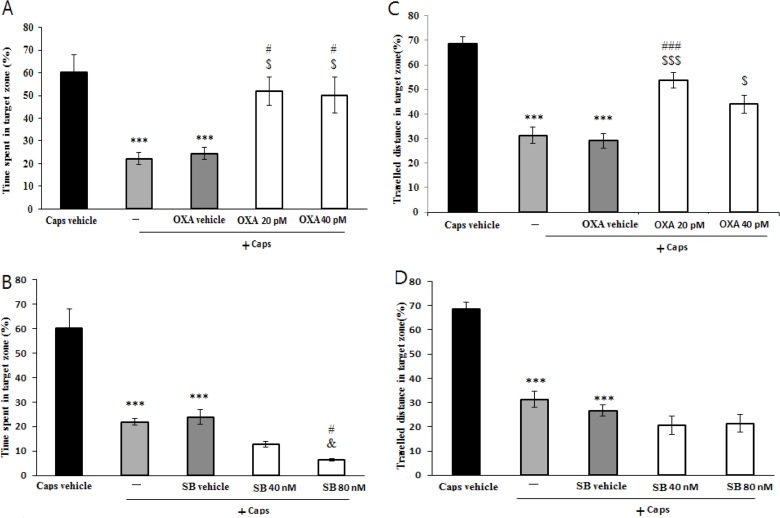
Retention performance was assessed in probe trial. Effects of pre-training intra-CA1 administration of orexin A and SB-334867-A on mean of time spent (A and B) and traveled distance (C and D) in capsaicin-treated rats (Caps) in the target region. Values correspond to mean±SEM (n=6), ***P<0.001 versus Caps vehicle group; ^###^P<0.001, ^#^P<0.05 versus Caps group, ^$$$^P<0.001, ^$$^P<0.0105 versus Caps+OXA vehicle; ^&^P<0.05 versus Caps+SB vehicle.

The results of probe trial are presented in Figure 4. The data showed that time spent (P<0.001) and the path length traveled (P<0.001) in the target region significantly reduced in rats treated with capsaicin as compared with that of vehicle group (Figure 4A and 4B). In addition, pretreatment with orexin A (20 and 40 pM) attenuated the effects of capsaicin on time spent in the target region (P<0.05) (Figure 4A). Likewise, orexin A (40 pM) significantly increased distance traveled in the target zone (P<0.01) (Figure 4B). As shown in Figure 4C, rats pretreated with SB-334867-A (80 nM/rat) prior to capsaicin administration, spent significantly less time in the target quadrat than capsaicin treatment group (P<0.05). Moreover, traveled distance decreased slightly, but not significantly in SB-334867-A (40 and 80 nM)-treated rats (Figure 4D).

## 4. Discussion

According to the current study, the capsaicin-treated animals that received intra-CA1 microinjection of orexin A showed a better performance in learning and memory tasks. Conversely, in rats pretreated with a selective OX1R antagonist SB-334867-A, learning, and memory efficiency were worse than those of the capsaicin treated rats. Pain-related cognitive dysfunction is examined in numerous rodent models of pain (Apkarian et al., 2004; Biessels et al., 1996; Cain, Francis, Plone, Emerich, & Lindner, 1997; Leite-Almeida et al., 2009; Raoof et al., 2016). Particularly, orofacial pain related learning, and memory dysfunction are recently noted. Rats’ MWM performance deficiency was reported following the tooth pulpal and intra-lip injection of capsaicin (Kooshki et al., 2016; Raoof et al., 2015b). Such phenomenon was related to hippocampal neuronal apoptosis and an increased Bax/Bcl2 ratio and elevated caspase 3 activity (Raoof et al., 2015a). However, the precise mechanism(s) of pain-induced memory deficiency is not fully clarified.

In the current study, CA1 administration of orexin A could attenuate spatial learning and memory deficiency in capsaicin treated rats. Orexin A-containing neurons are broadly distributed in several brain regions involved in the relay and modulation of nociceptive inputs (Peyron et al., 1998; Van Den Pol, 1999). It is extensively reported that orexin A has a modulatory effect on pain (Bingham et al., 2001; Ho et al., 2011). Surprisingly, previous studies showed that orexin A can modulate nociceptive transmission in the trigeminal complex mainly in Trigeminal Nucleus Caudalis (TNC) (Holland et al., 2006; Kooshki et al., 2016). In the current study, it is possible that the enhancing effect of orexin A on learning and memory is performed by modulation of hippocampal trigeminal nociceptive inputs (Burstein & Giesler, 1989).

It is shown that orexin neurons are innervated by neuropeptides releasing terminals including glutamate, gamma-aminobutyric acid (GABA), acetylcholine, nor-adrenaline, and serotoninergic (Rosin et al., 2003; Torrealba, Yanagisawa, & Saper, 2003; Yamanaka, Muraki, Tsujino, Goto, & Sakurai, 2003). Actually, OXRs activation might modify the secretion of other neurotransmitters co-localized with orexin terminals. Especially, orexin neurons coexist with glutamatergic neurons in various brain regions (Torrealba et al., 2003). It is shown that orexin modulates neural plasticity and potentiates N-methyl-D-aspartate (NMDA) receptor currents in the hippocampus through the release of norepinephrine, acetylcholine, and glutamate (Xia et al., 2009). Therefore, it could be suggested that orexin A plays a positive role in learning and memory function, partially mediated by increased glutamatergic currents. However, finding the detailed mechanisms of orexin on glutamate receptors signaling needs more and further investigations.

Chronic pain is commonly associated with overexpression of inflammatory and pro-apoptotic agents, which may increase the risk of neural inefficiency (Khairova, Machado-Vieira, Du, & Manji, 2009; Kozlovsky et al., 2007; Lucas, Rothwell, & Gibson, 2006). Particularly, inflammatory agents can suppress or disrupt the activity of orexinergic neurons (Gaykema and Goehler, 2009). It is shown that tumor necrosis factor has inhibitory effects on orexin neurons, predominately via degradation of orexin precursor mRNA (Zhan et al., 2011). Moreover, decrease in hippocampal expression of OX1Rs in orofacial pain situation is reported (Raoof et al., 2015b). In addition, orexin A anti-apoptotic and neuroprotective properties are reported in various cellular and molecular studies (Butterick, Nixon, Billington, 2012; Esmaeili-Mahani, Vazifekhah, Pasban-Aliabadi, Abbasnejad, & Sheibani, 2013; Kitamura et al., 2010). Therefore, it is plausible to assume that the possible neuroprotective features of orexin A may play an effective role to prevent learning and memory impairment in the current study. In conclusion, the results of the current study demonstrated that CA1-hippocampal OX1R may play a crucial role in pain-induced memory dysfunction.

## References

[B1] Akbari E. Motamedi F. Davoodi F. G. Noorbakhshnia M. Ghanbarian E. (2011). Orexin-1 receptor mediates long-term potentiation in the dentate gyrus area of freely moving rats. Behavioural Brain Research, 216(1), 375–80. doi: 10.1016/j.bbr.2010.08.017 20728473

[B2] Akbari E. Naghdi N. Motamedi F. (2006). Functional inactivation of orexin 1 receptors in CA1 region impairs acquisition, consolidation and retrieval in Morris Water Maze task. Behavioural Brain Research, 173(1), 47–52. doi: 10.1016/j.bbr.2006.05.028 16815564

[B3] Aou S. Li X. L. Li A. J. Oomura Y. Shiraishi T. Sasaki K. (2003). Orexin-A (hypocretin-1) impairs Morris Water Maze performance and CA1-Schaffer collateral long-term potentiation in rats. Neuroscience, 119(4), 1221–28. doi: 10.1016/s0306-4522(02)00745-5 12831875

[B4] Apkarian A. V. Sosa Y. Sonty S. Levy R. M. Harden R. N. Parrish T. B. (2004). Chronic back pain is associated with decreased prefrontal and thalamic gray matter density. The Journal of Neuro-science, 24(46), 10410–10415. doi: 10.1523/jneurosci.2541-04.2004 PMC6730296 15548656

[B5] Biessels G. J. Kamal A. Ramakers G. M. Urban I. J. Spruijt B. M. Erkelens D. W. (1996). Place learning and hippocampal synaptic plasticity in streptozotocin-induced diabetic rats. Diabetes, 45(9), 1259–1266. doi: 10.2337/diab.45.9.1259 8772732

[B6] Bingham S. Davey P. Babbs A. Irving E. Sammons M. Wyles M. (2001). Orexin-A, an hypothalamic peptide with analgesic properties. Pain, 92(1), 81–90. doi: 10.1016/s0304-3959(00)00470-x 11323129

[B7] Burstein R. Giesler G. J. (1989). Retrograde labeling of neurons in spinal cord that project directly to nucleus accumbens or the septal nuclei in the rat. Brain research, 497(1), 149–54. doi: 10.1016/0006-8993(89)90981-5 2790450

[B8] Butterick T. A. Nixon J. P. Billington C. J. Kotz C. M. (2012). Orexin A decreases lipid peroxidation and apoptosis in a novel hypothalamic cell model. Neuroscience Letters, 524(1), 30–34. doi: 10.1016/j.neulet.2012.07.002 22796468 PMC4467811

[B9] Cain C. K. Francis J. M. Plone M. A. Emerich D. F. Lindner M. D. (1997). Pain-related disability and effects of chronic morphine in the adjuvant-induced arthritis model of chronic pain. Physiology & Behavior, 62(1), 199–205. doi: 10.1016/s0031-9384(97)00158-3 9226363

[B10] Capra N. F. Dessem D. (1992). Central connections of trigeminal primary afferent neurons: Topographical and functional considerations. Critical Reviews in Oral Biology & Medicine, 4(1), 1–52. doi: 10.1177/10454411920040010101 1457683

[B11] Esmaeili-Mahani S. Vazifekhah S. Pasban-Aliabadi H. Abbasnejad M. Sheibani V. (2013). Protective effect of orexin-A on 6-hydroxydopamine-induced neurotoxicity in SH-SY5Y human dopaminergic neuroblastoma cells. Neurochemistry International, 63(8), 719–725. doi: 10.1016/j.neuint.2013.09.022 24135219

[B12] Gaykema R. P. Goehler L. E. (2009). Lipopolysaccharide challenge-induced suppression of Fos in hypothalamic orexin neurons: Their potential role in sickness behavior. Brain, Behavior, and Immunity, 23(7), 926–930. doi: 10.1016/j.bbi.2009.03.005 PMC2792632 19328847

[B13] Hart R. P. Martelli M. F. Zasler N. D. (2000). Chronic pain and neuropsychological functioning. Neuropsychology Review, 10(3), 131–49. doi: 10.1023/a:1009020914358 10983898

[B14] Ho Y. C. Lee H. J. Tung L. W. Liao Y. Y. Fu S. Y. Teng S. F. (2011). Activation of orexin 1 receptors in the periaqueductal gray of male rats leads to antinociception via retrograde endocanna-binoid (2-arachidonoylglycerol)-induced disinhibition. Journal of Neuroscience, 31(41), 14600–14610. doi: 10.1523/jneurosci.2671-11.2011 21994376 PMC3265563

[B15] Holland P. Akerman S. Goadsby P. (2006). Modulation of nociceptive dural input to the trigeminal nucleus caudalis via activation of the orexin 1 receptor in the rat. European Journal of Neuroscience, 24(10), 2825–2833. doi: 10.1111/j.1460-9568.2006.05168.x 17156207

[B16] Jaeger L. B. Farr S. A. Banks W. A. Morley J. E. (2002). Effects of orexin-A on memory processing. Peptides, 23(9), 1683–88. doi: 10.1016/s0196-9781(02)00110-9 12217429

[B17] Khairova R. A. Machado-Vieira R. Du J. Manji H. K. (2009). A potential role for pro-inflammatory cytokines in regulating synaptic plasticity in major depressive disorder. International Journal of Neuro-psychopharmacology, 12(4), 561–578. doi: 10.1017/s1461145709009924 PMC2771334 19224657

[B18] kitamura E. Hamada J. Kanazawa N. Yonekura J. Masuda R. Sakai F. (2010). The effect of orexin-A on the pathological mechanism in the rat focal cerebral ischemia. Neuroscience research, 68(2), 154–157. doi: 10.1016/j.neures.2010.06.010 20600373

[B19] Kooshki R. Abbasnejad M. Esmaeili-Mahani S. Raoof M. (2016). The role of trigeminal nucleus caudalis orexin 1 receptors in orofacial pain transmission and in orofacial induced learning and memory impairment in rats. Physiology & behavior, 157, 20–27. doi: 10.1016/j.physbeh.2016.01.031 26821188

[B20] Kozlovsky N. Matar M. A. Kaplan Z. Kotler M. Zohar J. Cohen H. (2007). Long-term down-regulation of BDNF mRNA in rat hippocampal CA1 subregion correlates with PTSD-like behavioural stress response. International Journal of Neuropsychopharmacology, 10(6), 741–758. doi: 10.1017/s1461145707007560 17291374

[B21] Leite-Almeida H. Almeida-Torres L. Mesquita A. R. Pertovaara A. Sousa N. Cerqueira J. J. (2009). The impact of age on emotional and cognitive behaviours triggered by experimental neuropathy in rats. Pain, 144(1), 57–65. doi: 10.1016/j.pain.2009.02.024 19398158

[B22] Lucas S. M. Rothwell N. J. Gibson R. M. (2006). The role of inflammation in CNS injury and disease. British Journal of Pharmacology, 147(1), 232–240. doi: 10.1038/sj.bjp.0706400 PMC1760754 16402109

[B23] Macfarlane T. V. Blinkhorn A. S. Davies R. M. Ryan P. Worthington H. V. Macfarlane G. J. (2002). Orofacial pain: Just another chronic pain? Results from a population-based survey. Pain, 99(3), 453–58. doi: 10.1016/s0304-3959(02)00181-1 12406520

[B24] Marcus J. N. Aschkenasi C. J. Lee C. E. Chemelli R. M. Saper C. B. Yanagisawa M. (2001). Differential expression of orexin receptors 1 and 2 in the rat brain. Journal of Comparative Neurology, 435(1), 6–25. doi: 10.1002/cne.1190 11370008

[B25] Moriarty O. McGuire B. E. Finn D. P. (2011). The effect of pain on cognitive function: A review of clinical and pre-clinical research. Progress in Neurobiology, 93(3), 385–404. doi: 10.1016/j.pneurobio.2011.01.002 21216272

[B26] Morris R. (1984). Development of a water-maze procedure for studying spatial learning in the rat. Journal of Neuroscience Methods, 11(1), 47–60. doi: 10.1016/0165-0270(84)90007-4 6471907

[B27] Peyron C. Tighe D. K. Van Den Pol A. N. De Lecea L. Heller H. C. Sutcliffe J. G. (1998). Neurons containing hypocretin (orexin) project to multiple neuronal systems. Journal of Neuroscience, 18(23), 9996–10015. PMID: 9822755 10.1523/JNEUROSCI.18-23-09996.1998 PMC6793310

[B28] Raoof M. Ebrahimnejad H. Abbasnejad M. Amirkhosravi L. Raoof R. Esmaeili Mahani S. (2016). The effects of inflammatory tooth pain on anxiety in adult male rats. Basic and Clinical Neuroscience, 7(3), 259–268. doi: 10.15412/j.bcn.03070311 27563419 PMC4981838

[B29] Raoof M. Esmaeili-Mahani S. Nourzadeh M. Raoof R. Abbasnejad M. Amirkhosravi L. (2015). Noxious stimulation of the rat tooth pulp may impair learning and memory through the induction of hippocampal apoptosis. Journal of Oral & Facial Pain and Headache, 29(4), 390–7. doi: 10.11607/ofph.1452. 26485387

[B30] Raoof R. Esmaeili-Mahani S. Abbasnejad M. Raoof M. Sheibani V. Kooshki R. (2015). Changes in hippocampal orexin 1 receptor expression involved in tooth pain-induced learning and memory impairment in rats. Neuropeptides, 50, 9–16. doi: 10.1016/j.npep.2015.03.002 25817882

[B31] Rosin D. L. Weston M. C. Sevigny C. P. Stornetta R. L. Guyenet P. G. (2003). Hypothalamic orexin (hypocretin) neurons express vesicular glutamate transporters VGLUT1 or VGLUT2. Journal of Comparative Neurology, 465(4), 593–603. doi: 10.1002/cne.10860 12975818

[B32] Sakurai T. Amemiya A. Ishii M. Matsuzaki I. Chemelli R. M. Tanaka H. (1998). Orexins and orexin receptors: A family of hypothalamic neuropeptides and G protein-coupled receptors that regulate feeding behavior. Cell, 92(4), 573–85. doi: 10.1016/s0092-8674(00)80949-6 9491897

[B33] Sessle B. J. (1999). Neural mechanisms and pathways in craniofacial pain. Canadian Journal of Neurological Sciences, 26(3), 7–11. doi: 10.1017/s0317167100000135 10563227

[B34] Shu Q. Hu Z. L. Huang C. Yu X. W. Fan H. Yang J. W. (2014). Orexin-A promotes cell migration in cultured rat astrocytes via Ca^2+^-dependent PKCα and ERK1/2 signals. PLoS ONE, 9(4), 95259. doi: 10.1371/journal.pone.0095259 PMC3991588 24748172

[B35] Torrealba F. Yanagisawa M. Saper C. (2003). Colocalization of orexin a and glutamate immunoreactivity in axon terminals in the tuberomammillary nucleus in rats. Neuroscience, 119(4), 1033–44. doi: 10.1016/s0306-4522(03)00238-0 12831862

[B36] Trivedi P. Yu H. MacNeil D. J. van der Ploeg L. Guan X. M. (1998). Distribution of orexin receptor mRNA in the rat brain. FEBS letters, 438(1–2), 71–75. doi: 10.1016/s0014-5793(98)01266-6 9821961

[B37] van Den Pol A. N. (1999). Hypothalamic hypocretin (orexin): Robust innervation of the spinal cord. Journal of Neuroscience, 19(8), 3171–82. PMID: 10191330 10.1523/JNEUROSCI.19-08-03171.1999 PMC6782271

[B38] Wayner M. Armstrong D. Phelix C. Oomura Y. (2004). Orexin-A (Hypocretin-1) and leptin enhance LTP in the dentate gyrus of rats in vivo. Peptides, 25(6), 991–6. doi: 10.1016/j.peptides.2004.03.018 15203246

[B39] Xia J. Chen F. Ye J. Yan J. Wang H. Duan S. (2009). Activity-dependent release of adenosine inhibits the glutamatergic synaptic transmission and plasticity in the hypothalamic hypocretin/orexin neurons. Neuroscience, 162(4), 980–8. doi: 10.1016/j.neuroscience.2009.05.033 19465088

[B40] Yamamoto T. Saito O. Shono K. Aoe T. Chiba T. (2003). Anti-mechanical allodynic effect of intrathecal and intracerebroventricular injection of orexin-A in the rat neuropathic pain model. Neuroscience Letters, 347(3), 183–6. doi: 10.1016/s0304-3940(03)00716-x 12875916

[B41] Yamanaka A. Muraki Y. Tsujino N. Goto K. Sakurai T. (2003). Regulation of orexin neurons by the monoaminergic and cholinergic systems. Biochemical and Biophysical Research Communications, 303(1), 120–9. doi: 10.1016/s0006-291x(03)00299-7 12646175

[B42] Zhan S. Cai G. Q. Zheng A. Wang Y. Jia J. Fang H. (2011). Tumor necrosis factor-alpha regulates the Hypocretin system via mRNA degradation and ubiquitination. Biochimica et Biophysica Acta (BBA)-Molecular Basis of Disease, 1812(4), 565–71. doi: 10.1016/j.bbadis.2010.11.003 21094253 PMC3042489

